# Stimulating cingulate: distinct behaviours arise from discrete zones

**DOI:** 10.1093/brain/awy224

**Published:** 2018-09-25

**Authors:** Matthew A J Apps

**Affiliations:** 1 Department of Experimental Psychology, University of Oxford, UK; 2 Wellcome Centre for Integrative Neuroimaging, University of Oxford, UK

## Abstract

This scientific commentary refers to ‘Motor and emotional behaviours elicited by electrical stimulation of the human cingulate cortex’, by Caruana *et al.*. (doi:10.1093/brain/awy219).

This scientific commentary refers to ‘Motor and emotional behaviours elicited by electrical stimulation of the human cingulate cortex’, by Caruana *et al.*. (doi:10.1093/brain/awy219).

The cingulate cortex is one of the most commonly described loci of dysfunction in psychiatry and neurology, and as regions of the brain go, its functional properties have stirred considerable controversy amongst neuroscientists (Ebitz and Hayden, 2016). Such debates have even led to the region having its own social media hashtag (#cingulategate). Why such controversy? The answer to this is multifaceted but two key factors are (i) most of the theoretical accounts of cingulate function are based on informative but correlational neuroimaging data; and (ii) although appearing by eye to be a continuous piece of tissue, the cingulate cortex in fact comprises discrete anatomical zones, each of which may have distinct functional properties (Vogt, 2009). In this issue of *Brain*, Caruana and co-workers provide exactly the causal data that can begin to resolve such issues, take the field beyond current impasses, and provide new insights into the functional organization of this passionately debated section of grey matter (Caruana *et al.*, 2018).

Caruana *et al.* recorded the behaviours and self-reported affective states arising from electrical stimulation of the cingulate cortices in 329 patients with epilepsy. Although testing the consequences of electrical stimulation is common in patients with drug-resistant epilepsy and has been used to probe cingulate function before (Parvizi *et al.*, 2013), Caruana and colleagues provide a unique and substantial dataset, with a total of 1789 stimulation sites. Unlike previous studies, these sites were located across the whole cingulate cortex (Fig. 1), from the most posterior to the most anterior portions, and with stereo-electroencephalography (SEEG) electrodes precisely localized using a multimodal approach. This breadth of sites allowed the authors to go beyond previous work and adopt a localization-based approach more akin to a neuroimaging study. But rather than finding correlated brain signals, they were able to identify the self-reported ‘feeling’ or type of movement elicited by causal manipulation of different regions of the cingulate cortex. In doing so, they obtained results that both converge nicely with current knowledge of the anatomical and functional organization of the region, but also results that do not fit with many popular theories for specific cingulate subregions.

**Figure 1:**
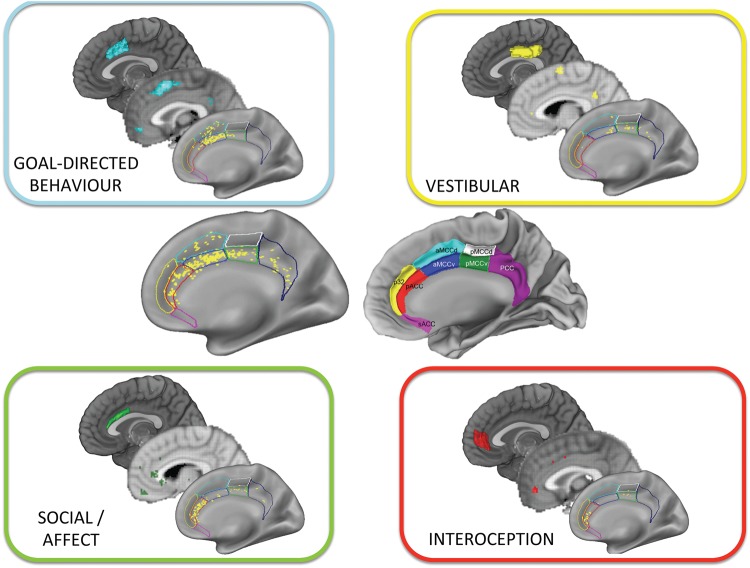
**Discrete functions elicited by electrical stimulation of different cingulate cortex zones.** *Centre left* shows the location of all stimulation sites in Caruana *et al.* (2018). *Centre right* shows the putative anatomical zones of the cingulate cortex (both images taken from Caruana *et al.*, 2018). Within each box is the location of sites that elicited the responses from participants (*bottom*), the thresholded (Z = 5) forward inference map (*middle*) from functional MRI meta-analysis tool neurosynth.org for the closest possible corresponding terms (‘goal directed’, ‘affective’, ‘interoceptive’, ‘balance’), and the region of the cingulate cortex as identified by neuroimaging through connectivity-based parcellation of cingulate cortex (*top*) with images created from masks developed by Neubert *et al.* (2015).

Although many functions have been ascribed to the cingulate cortex, there is evidence that this area consists of discrete cytoarchitectonic zones, with each having a different profile of connections to other brain areas and distinct functional properties (Vogt, 2009; Neubert *et al.*, 2015). Broadly speaking the cingulate can be divided into anterior, mid, and posterior (ACC, MCC, PCC) zones, with further subregions within them (Fig. 1). Caruana and colleagues’ results show striking parallels of such anatomical organization in the behaviours evoked by stimulation. The most ventral-anterior portions in pregenual ACC evoked interoceptive sensations (e.g. sensations in the gut). Stimulation of the area slightly posterior to this in the perigenual ACC, predominantly lying in the gyral portion, caused participants to engage in the social-emotional process of laughing. Posterior to this, in the MCC, stimulation led to goal-directed movements, whereas vestibular sensations were evoked in the PCC (Fig. 1).

Why is the localization of these different functions to distinct zones important? Often clinical and cognitive neuroscience characterizations operate under the assumption that functions ascribed to the cingulate cortex are processed within the same brain region. This is clearly not the case. Previous neuroimaging research has shown activity in different regions of cingulate cortex for interoceptive, social and goal-directed information processing (Critchley *et al.*, 2004; Apps *et al.*, 2016; Kolling *et al.*, 2016), but here Caruana and colleagues provide compelling causal evidence to support these accounts previously based largely on correlational imaging data. For cognitive neuroscience, this re-emphasizes the importance of precise localization for making inferences about function and when debating theories. Some current debates may in fact have arisen because theories that are believed to be competing accounts of the same region, are in fact accounts of two different cingulate subregions and thus should not be considered as competing at all. For clinical neuroscience, it highlights how crucial it is to understand which particular subregion of the cingulate cortex dysfunction is localized to. With these functions in such close proximity to each other, the mechanisms underlying disease, the targeting of specific circuits by treatments, and the prediction of symptoms will all hinge on precise localization.

Not only do Caruana *et al.* point to the need to localize function accurately, some of their findings question whether prominent theories of one subregion are sufficient for explaining evoked behaviours. The anterior portions of the MCC (aMCC) correspond to what is often termed dorsal anterior cingulate cortex (dACC), with some theories suggesting that this area plays a crucial role in pain processing. Caruana and colleagues found that stimulation of the aMCC was highly likely to evoke movement. There was, however, little evidence of any nociceptive effects in this subregion. This raises questions for theories that it is part of the ‘pain matrix’ and crucial for processing information about pain. Beyond this it certainly provides rather robust evidence that this subregion is not ‘selective for pain’ as some have argued (see Ebitz and Hayden, 2016 for discussion).

The movements evoked by MCC/dACC stimulation were also not just muscular innervations, they were goal-directed. This aligns with many theories that posit a role for this area in goal-directed behaviour, motivation and cognitive control (Kolling *et al.*, 2016; Le Heron *et al.*, 2017). A previous stimulation study by Parvizi and colleagues (2013) showed that MCC stimulation evoked a feeling of a ‘will to persevere’. Taken together with Caruana *et al.* (2018) these findings would support the notion that aMCC might be engaged when energizing and motivating goal-directed behaviours. However, what was more surprising was that even within the aMCC there were still differences in the nature of evoked behaviour. Extending stimulation along the dorso-ventral axis led to different movement goals. Stimulation of dorsal aMCC evoked movements directed towards the body, but ventral stimulation led to whole body movements towards extrapersonal space (e.g. the impulse to get up). Such findings cannot be readily explained within current cognitive control or motivational accounts of the aMCC.

Could theories of the role of the aMCC in motivation and cognitive control be adapted to account for such organization of different types of movement? One possibility is that the whole of the aMCC is involved in these processes, but motivation and cognitive control are organized within a space- or body-framed topography. That is, similar computations are performed for movements across the region, but specific types of goal-directed actions are coded by different subregions within the MCC. Support for such embodied representation has been shown in human neuroimaging studies, with movements of different parts of the body evoking responses in different locations even within the MCC (Procyk *et al.*, 2016). Going further than this, there is also evidence to suggest this embodiment is not just tied to the movements but could be related to cognitive control and to motivationally relevant information that guides them. Neurophysiological studies in a homologous region of the macaque monkey show that responses elicited by feedback occur in distinct zones within the MCC when the feedback relates to a different part of the body (e.g. eye or limb) (Procyk *et al.*, 2016). Cognitive control and motivation may therefore be embodied within the MCC in a manner that has traditionally been thought of as the preserve of lower-level features of movements in other motor regions. Such a notion will likely require current cognitive and computational theories of the aMCC/dACC to be updated, but also opens up possibilities for new ways of conceptualizing disorders of goal-directed behaviour linked to this region (Le Heron *et al.*, 2017).

Neuroscience is moving at an ever more rapid pace, towards ever more sophisticated neuroimaging approaches. Caruana *et al.* provide an important, timely reminder of how precise localization is still crucial and how much causal methods still have to offer. This most comprehensive of stimulation studies gives considerable insights, providing affirmation for existing neuroimaging findings, as well as straining and constraining theoretical accounts of cingulate function. In doing so it is likely to stimulate still further debate.

**Glossary**

**Anterior cingulate cortex (ACC):** The most anterior zone of the cingulate cortex extending into the frontal cortex. It includes a subgenual (sACC) subregion lying below the corpus callosum, a pregenual section, which lies superior to (above) the sACC and a perigenual subregion lying dorsal and posterior to (above and behind) that. Pregenual stimulation elicited laughter, perigenual elicited interoceptive sensations.

**Midcingulate cortex (MCC)**: The zone of the cingulate cortex lying posterior to the ACC. It contains at least four subregions, two of which are anterior to the others but lie posterior to the ACC, one being more dorsal (aMCCd) to the other (aMCCv), with two further subregions directly posterior to those (pMCCd and pMCCv). These regions are often referred to as dorsal anterior cingulate cortex (dACC). Goal-directed behaviours were elicited by stimulation of aMCC.

**Posterior cingulate cortex (PCC):** The zone of the cingulate cortex lying caudal to the other zones. Stimulation elicited vestibular sensations.

**Stereo-electroencephalography electrode localization:** To precisely determine the location of electrodes, patients underwent magnetic resonance scans after electrode implantation. The electrode locations are warped onto a template brain allowing for comparison of stimulation sites within a unified space across patients. This allows greater precision for localization of electrodes, and for them to be compared to previous anatomical parcellations of the cingulate cortex.
